# Draft genome of *Rosenbergiella nectarea* strain 8N4^T^ provides insights into the potential role of this species in its plant host

**DOI:** 10.7717/peerj.8822

**Published:** 2020-04-06

**Authors:** Sivan Laviad-Shitrit, Ido Izhaki, William B. Whitman, Nicole Shapiro, Tanja Woyke, Nikos C. Kyrpides, Malka Halpern

**Affiliations:** 1 Department of Evolutionary and Environmental Biology, Faculty of Natural Sciences, University of Haifa, Haifa, Israel; 2 Department of Microbiology, University of Georgia, Athens, GA, USA; 3 Department of Energy Joint Genome Institute, DOE Joint Genome Institute, Berkeley, CA, USA; 4 Department of Biology and Environment, Faculty of Natural Sciences, University of Haifa, Oranim, Kiryat Tivon, Israel

**Keywords:** *Rosenbergiella nectarea*, Flower, Nectar, Whole genome, Attractant, Repellant, Pectinase, Pyocin

## Abstract

**Background:**

*Rosenbergiella nectarea* strain 8N4^T^, the type species of the genus *Rosenbergiella*, was isolated from *Amygdalus communis* (almond) floral nectar. Other strains of this species were isolated from the floral nectar of *Citrus paradisi* (grapefruit), *Nicotiana glauca* (tobacco tree) and from *Asphodelus aestivus. R. nectarea* strain 8N4^T^ is a Gram-negative, oxidase-negative, facultatively anaerobic bacterium in the family *Enterobacteriaceae*.

**Results:**

Here we describe features of this organism, together with its genome sequence and annotation. The DNA GC content is 47.38%, the assembly size is 3,294,717 bp, and the total number of genes are 3,346. The genome discloses the possible role that this species may play in the plant. The genome contains both virulence genes, like pectin lyase and hemolysin, that may harm plant cells and genes that are predicted to produce volatile compounds that may impact the visitation rates by nectar consumers, such as pollinators and nectar thieves.

**Conclusions:**

The genome of *R. nectarea* strain 8N4^T^ reveals a mutualistic interaction with the plant host and a possible effect on plant pollination and fitness.

## Introduction

Floral nectar is a unique and harsh environment that contains mainly sugar (up to 90% of dry weight) and other compounds, such as amino acids, organic acids, lipids, essential oils, polysaccharides, vitamins, antioxidants, minerals and secondary metabolites (Fridman et al., 2012). Floral nectar is regarded as a reward and a key component between animal-pollinated plants and their pollinators (Brandenburg et al., 2009). Gilliam, Moffett & Kauffeld (1983) and Ehlers & Olesen (1997) were the first who found indications for the presence of microorganisms in floral nectar. It took 15 years before more detailed studies regarding the identity of the bacterial communities in the nectar of different plant species was published (Fridman et al., 2012; Álvarez-Pérez & Herrera, 2013). Since then, intensive research was conducted in this specific ecological niche and the data and publications are accumulating at an exponential rate.

*Rosenbergiella nectarea* strain 8N4^T^ (=LMG 26121^T^ =DSM 24150^T^) is the type species of the genus *Rosenbergiella* and was isolated from floral nectar of *Amygdalus communis* (almond) in northern Israel (Fridman et al., 2012; Halpern et al., 2013). *R. nectarea* strains were also isolated by Fridman et al. (2012) and Samuni-Blank et al. (2014) from floral nectar of *Nicotiana glauca* (tobacco tree), *Citrus paradisi* (grapefruit) and *Asphodelus aestivus*. The prevalence of *R. nectarea* strains out of the total cultured floral nectar microbiota in floral nectar of almond, tobacco, grapefruit and *A. aestivus* were 41.2%, 12.5%, 42.0% and 4.5%, respectively (Fridman et al., 2012; Samuni-Blank et al., 2014).

The genus *Rosenbergiella* is a member in the family *Enterobacteriaceae* and currently includes four species: the *R. nectarea* strain 8N4^T^ (the type species), which was isolated and identified by Halpern et al. (2013) and *R. australiborealis*, *R. collisarenosi* and *R. epipactidis*, which were isolated and identified by Lenaerts et al. (2014). Lenaerts et al. (2014) isolated *Rosenbergiella* species from the floral nectar of different plant species in different countries: *Epipactis palustris* (Belgium and France), *Iris xiphium* (Spain), *Narcissus papyraceus* (Spain) and *Protea roupelliae* and *P. subvestita* (South Africa).

Here we describe the features of *R. nectarea* strain 8N4^T^ together with the analyses and annotation of the strain’s draft genome sequence. The genome reveals the possible role that *R. nectarea* may play in its host. The genome contains both virulence genes that may harm plant cells and genes that may produce volatile compounds, which may impact visitation rates by nectar consumers, such as pollinators and nectar thieves.

## Materials and Methods

The draft genome of *R. nectarea* strain 8N4^T^ was generated at the US Department of Energy (DOE), Joint Genome Institute (JGI) (jgi.doe.gov). This project was part of the Genomic Encyclopedia of Type Strains, Phase III (KMG-III): the genomes of soil and plant-associated and newly described type strains (Whitman et al., 2015).

### Genomic DNA preparation

*Rosenbergiella nectarea* strain 8N4^T^ was grown in Luria broth (LB) (HiMedia, Mumbai, India) supplemented with 10% sucrose at 28 °C. Cells were harvested by centrifugation and genomic DNA was extracted using a DNA isolation kit (DNeasy Blood & Tissue Kit, Qiagen, Germany), according to the manufacturer’s instructions. The purity of the genomic DNA preparation was assessed by PCR amplification and partial sequencing of the 16S rRNA gene. The pure genomic DNA was shipped to DOE, JGI for genome sequencing.

### Genome sequencing and assembly

The draft genome of *R. nectarea* strain 8N4^T^ was generated using Illumina technology (Bennett, 2004). In order to construct the Illumina library, 200 ng of DNA was sheared to 300 bp using the Covaris LE220 (Covaris, Woburn, MA, USA) and size selected using SPRI beads (Beckman Coulter, Brea, CA, USA). The fragments were treated with end-repair, A-tailing and ligation of Illumina compatible adapters (IDT, Inc., San Diego, CA, USA) using the KAPA-Illumina library creation kit (KAPA Biosystems, Wilmington, MA, USA). The prepared library was quantified using KAPA Biosystem’s next-generation sequencing library qPCR kit and run on a Roche LightCycler 480 real-time PCR instrument. The quantified library was then multiplexed with other libraries, and the pool of libraries was then prepared for sequencing on the Illumina HiSeq sequencing platform utilizing a TruSeq paired-end cluster kit, v4 and Illumina’s cBot instrument to generate a clustered flow cell for sequencing. Sequencing of the flow cell was performed on the Illumina HiSeq 2500 sequencer using HiSeq TruSeq SBS sequencing kits, v4, following a 2 × 150 indexed run recipe. The Illumina HiSeq platform generated 3,294,717 reads and 867 Mb. Reads with more than one “N”, or with quality scores (before trimming) averaging less than 8, or reads shorter than 51 bp (after trimming), were discarded. Remaining reads were mapped to masked versions of human, cat and dog references using BBMAP (BBTools version 35.82) (http://sourceforge.net/projects/bbmap), and discarded if identity exceeded 95%. Sequence masking was performed with BBMask (BBTools version 35.82) (http://sourceforge.net/projects/bbmap). The following steps were then performed for assembly: (1) artifact-filtered Illumina reads were assembled using SPAdes (version 3.6.2) (Bankevich et al., 2012); (2) assembly contigs were discarded if the length was <1 kbp. Parameters for the SPAdes assembly were: —cov–cutoff auto—phred–offset 33 –t 8 –m 40—careful–k 25,55,95—12.

### Genome annotation

Protein-coding genes were identified using Prodigal version 2.50 (Hyatt et al., 2010), as part of the DOE-JGI genome-annotation pipeline (Huntemann et al., 2015; Chen et al., 2016). Additional gene prediction analysis and manual functional annotation were performed within the Integrated Microbial Genomes platform, which provided tools for analyzing and reviewing the structural and functional annotations of genomes in a comparative context (Chen et al., 2017). Table 1 summarizes the genome product information.

**Table 1:** Genome sequencing project information.

MIGS ID*	Property	Term
MIGS 31.1	Finishing quality	High quality draft
MIGS 28	Libraries methods	Illumina regular fragment, 300 bp, tubes
MIGS 29	Sequencing platform	Illumina HiSeq 2500-1TB
MIGS 31.2	Fold coverage	263x
MIGS 30	Assembly method	ALLPATHS v. r46652
MIGS 32	Gene-calling method	Prodigal
	Locus tag	A8730
	GenBank ID	FOGC00000000
	GenBank date of release	3 September 2016
	GOLD ID	Gp0131806
	BIOPROJECT	PRJNA322879
MIGS 13	Source material identifier	DSM 24150^T^
	Project relevance	KMG-III: genomes of plant-associated type strains

**Note:**

* MIGS, Minimum Information about a Genome Sequence (Field et al., 2008).

### Electron microscopy

For electron microscopy, we used a JEM-1200EX electron microscope (JEOL). Bacterial cells were grown on both LB agar (HiMedia, Mumbai, India) and LB agar supplemented with 10% sucrose for 48 h at 28 °C and were then suspended in saline water (0.85% NaCl). Samples were fixed to a carbon-coated grid and stained with 2% uranyl acetate; they were then photographed using the JEOL microscope mentioned above.

## Results

**Figure 1:**
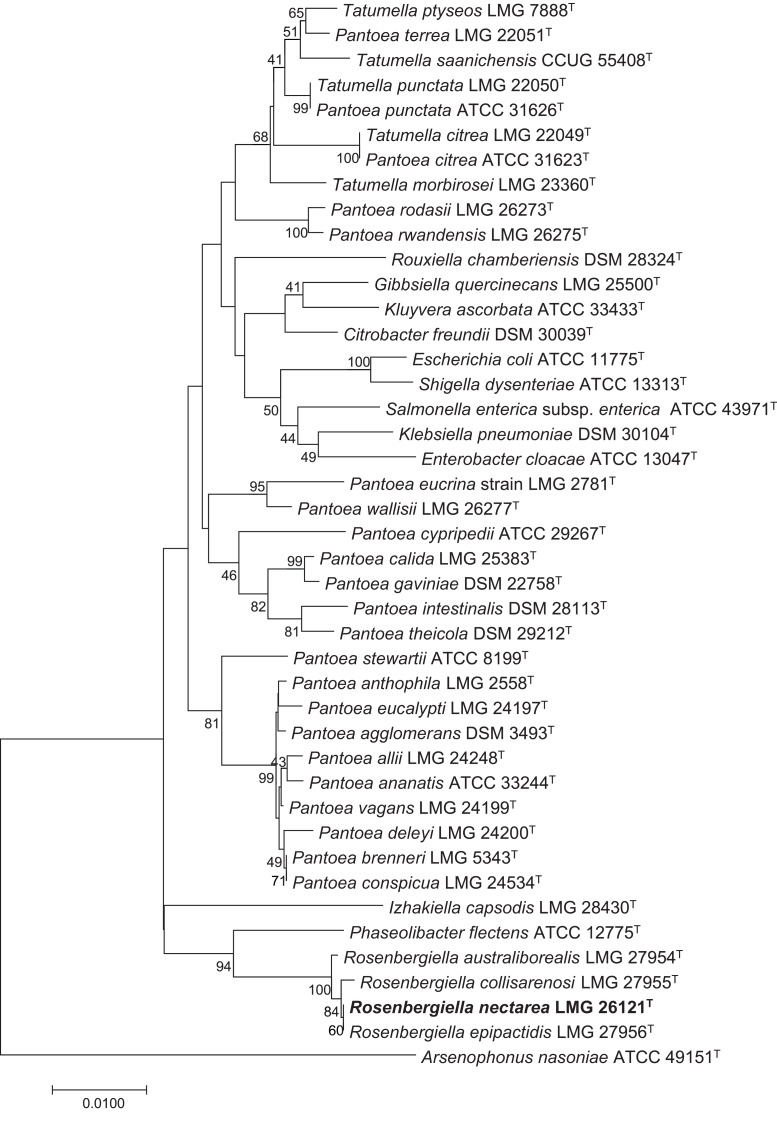
Phylogenetic tree based on 16S rRNA gene sequences, highlighting the position of *R. nectarea* strain 8N4^T^ relative to type species within the order *Enterobacteriales*. The sequence alignments were performed using the CLUSTAL W program and the tree was generated using the neighbor-joining method in MEGA 5 software. The nucleotide substitution model used in the analysis was Maximum Composite Likelihood. Bootstrap values (from 1,000 replicates) greater than 40% are shown at the branch points. The bar indicates 0.01 substitutions per nucleotide position. ****

**Figure 2:**
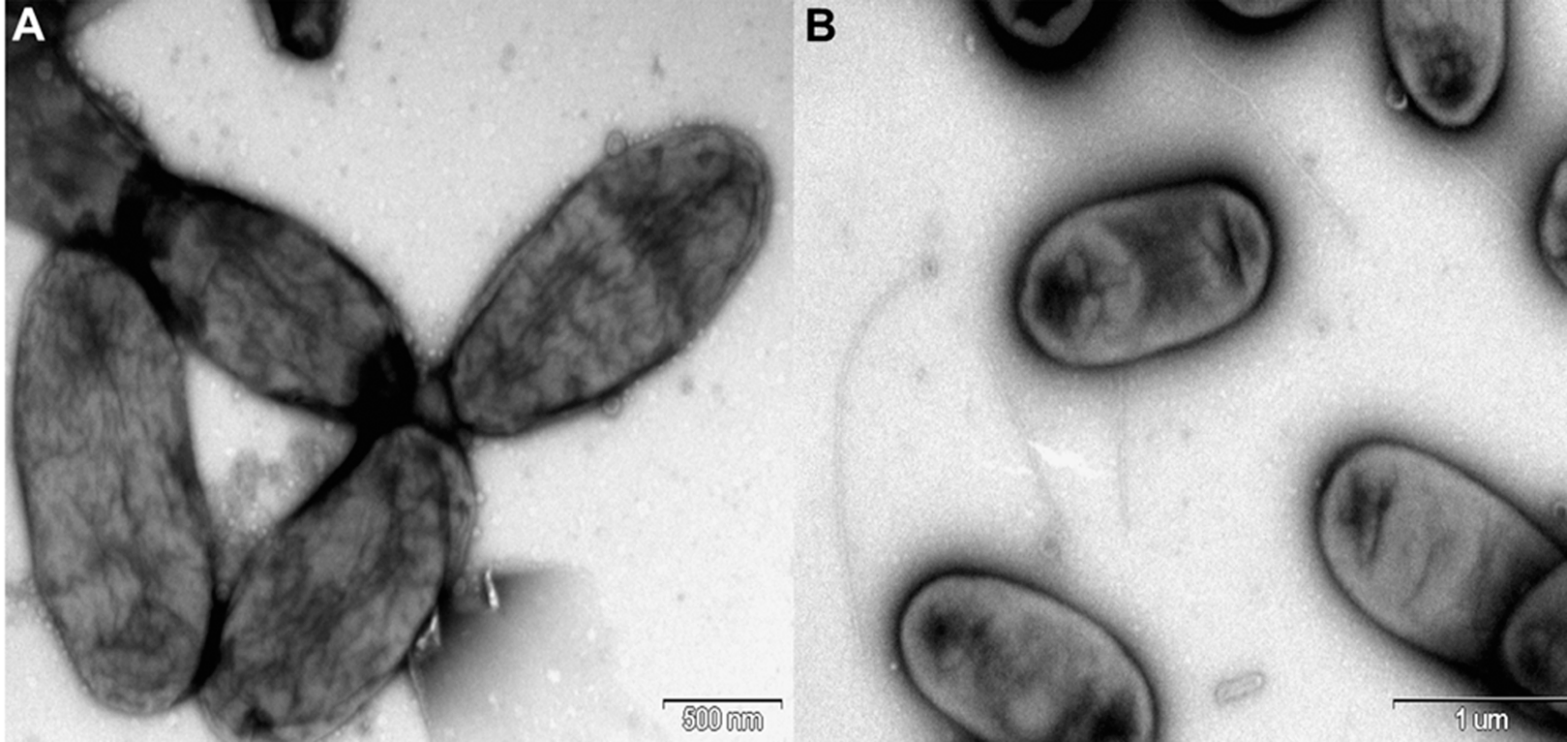
Electron micrograph of negatively stained cells of *R. nectarea* strain 8N4^T^. Cells are nonflagellated rods when grown on culture media supplemented with sucrose. (A) However, cells with a flagellum can be seen when the strain is grown on culture media without the supplementation of sucrose. (B) Bar, 0.5 and 1.0 µm (A and B, respectively).

*Rosenbergiella nectarea* strain 8N4^T^ belongs to the *Enterobacteriaceae* family. A neighbor-joining tree based on 16S rRNA gene sequences shows that its closest relatives are plant-associated species: *Rosenbergiella*, *Phaseolibacter* and *Izhakiella* (Lenaerts et al., 2014; Aizenberg-Gershtein et al., 2016a, 2016b) (Fig. 1). Electron-microscope imaging enabled the measurement of strain 8N4^T^ cells. *R. nectarea* strain 8N4^T^ is rod-shaped and variable in size (0.6–1.1 mm wide and 1.2–3.0 mm long). Interestingly, the bacterial cells are nonflagellated rods when grown on culture media supplemented with sucrose (Fig. 2A). However, when cells are grown on media without sucrose supplementation, flagella can be observed (Fig. 2B).

After 48 hours of incubation on LB agar supplemented with 10% sucrose at 28 °C under aerobic conditions, colonies of *R. nectarea* strain 8N4^T^ are circular and smooth with a yellow/orange pigment. This species demonstrates poor growth in the absence of sucrose. The strain grows at 4–35 °C (optimum 28–30 °C), with 0.0–5.0% (w/v) NaCl (optimum 3% NaCl) and with 0–60% sucrose (optimum 10–25% sucrose) (Halpern et al., 2013). Table 2 shows a summary of the strain’s classification and general features.

**Table 2:** Classification and general features of *R. nectarea* strain 8N4^T^ according to Minimum Information about a Genome Sequence (MIGS) recommendations (Field et al., 2008).

MIGS ID	Property	Term	Evidence code^a^
	Classification	Domain *Bacteria*	TAS (Woese, Kandler & Wheelis, 1990)
		Phylum *Proteobacteria*	TAS (Garrity & Lilburn, 2005)
		Class *Gammaproteobacteria*	TAS (Garrity, Holt & Lilburn, 2005)
		Order *Enterobacterales*	TAS (Garrity, 2001)
		Family *Enterobacteriaceae*	TAS (Octavia & Lan, 2014)
		Genus *Rosenbergiella*	TAS (Halpern et al., 2013)
		Species *Rosenbergiella nectarea*	TAS (Halpern et al., 2013)
		Type strain DSM 24150^T^	TAS (Halpern et al., 2013)
	Gram stain	Negative	TAS (Halpern et al., 2013)
	Cell shape	Rod-shaped	TAS (Halpern et al., 2013)
	Motility	Motile	TAS (Halpern et al., 2013)
	Sporulation	Non-sporulating	IDS
	Temperature range	4–35 °C	TAS (Halpern et al., 2013)
	Optimum temperature	28–30 °C	TAS (Halpern et al., 2013)
	Energy metabolism	Chemoheterotrophic	TAS (Halpern et al., 2013)
	Carbon source	Glucose	TAS (Halpern et al., 2013)
MIGS-6	Habitat	Floral nectar	TAS (Halpern et al., 2013)
MIGS-6.3	Salinity	Halotolerant	TAS (Halpern et al., 2013)
MIGS-22	Oxygen requirement	Facultative anaerobic	TAS (Halpern et al., 2013)
MIGS-15	Biotic relationship	Free living, plant host-associated	TAS (Halpern et al., 2013)
MIGS-14	Pathogenicity	Non-pathogenic	NAS
MIGS-4	Geographic location	Northern Israel	TAS (Halpern et al., 2013)
MIGS-5	Collection date	March–June 2009	TAS (Halpern et al., 2013)

**Note:**

a Evidence codes: IDA, Inferred from Direct Assay; TAS, Traceable Author Statement (i.e., a direct report exists in the literature); NAS, Non-traceable Author Statement (i.e., not directly observed for the living, isolated sample but based on a generally accepted property for the species or anecdotal evidence). Evidence codes are from the Gene Ontology project (Ashburner et al., 2000).

### Genome properties

**Figure 3:**
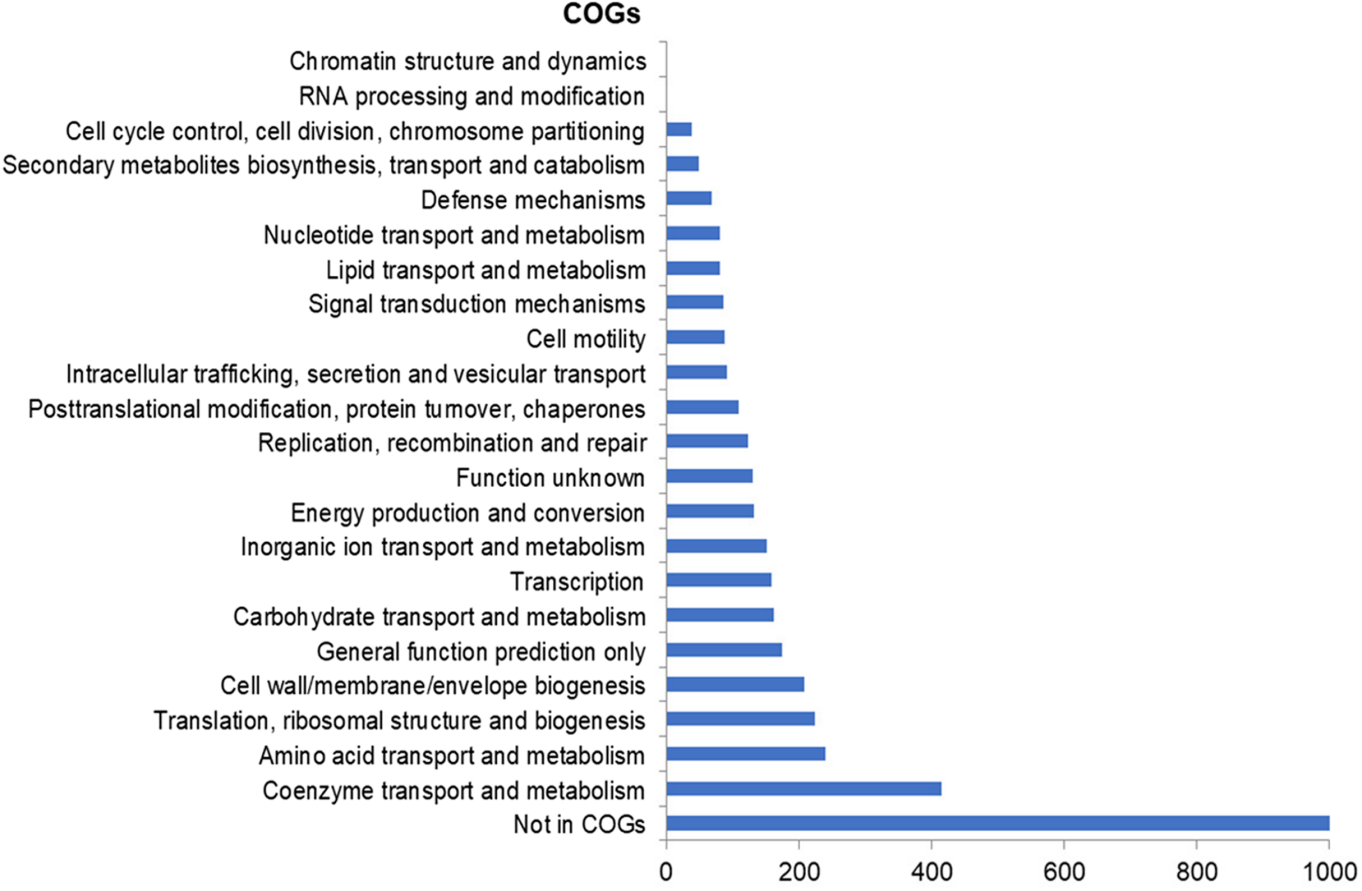
Number of genes assigned to COG categories for *R. nectarea* strain 8N4^T^.

**Figure 4:**
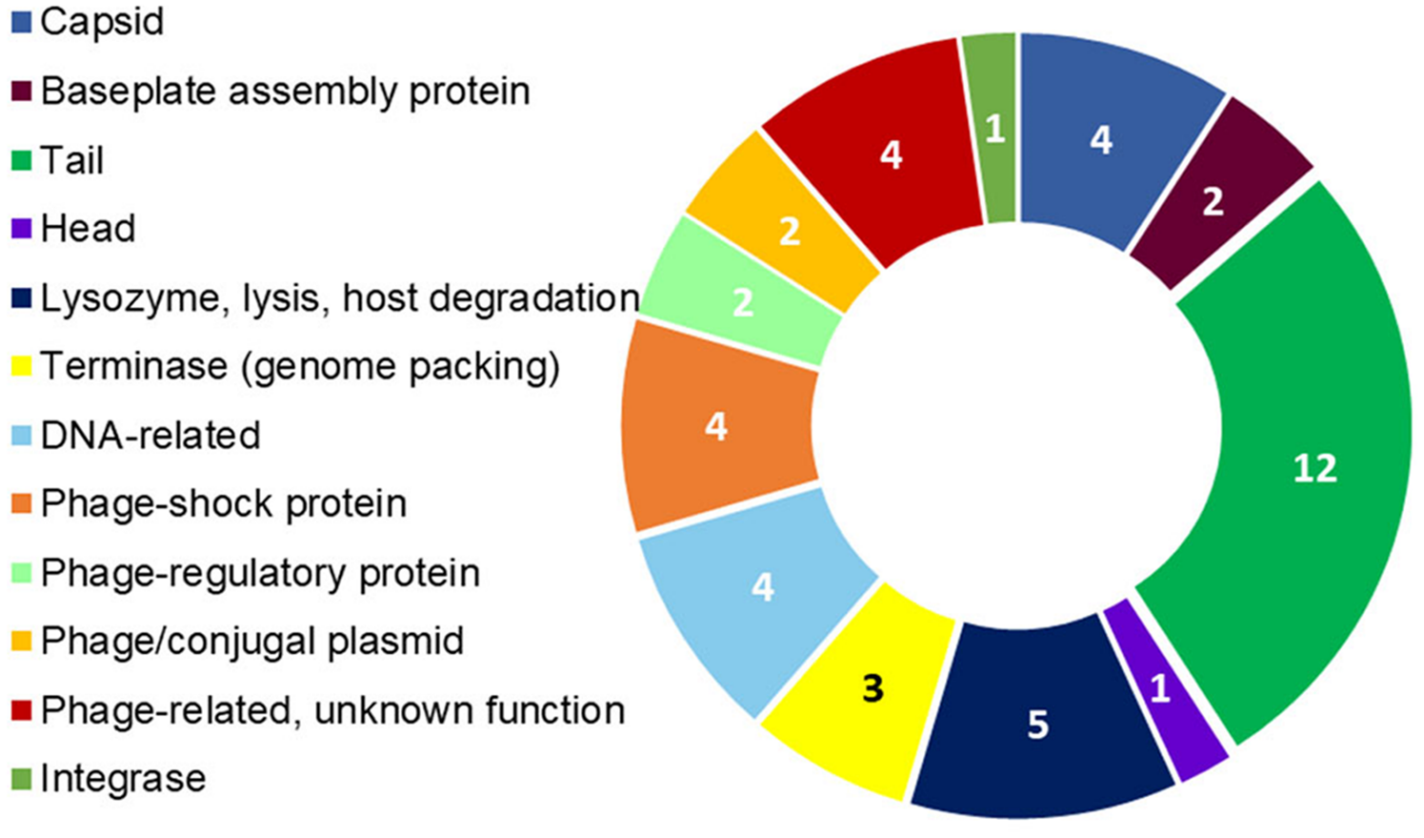
*R. nectarea* strain 8N4^T^ prophage genes. The presence of 44 genes of a prophage within the genome of *R. nectarea* (see also Table S1). The figures in the diagram represent the number of genes counted.

The assembly of the draft genome sequence resulted in 30 scaffolds amounting to 3,294,717 bp, and the GC content was 47.38% (Table 3). The number of predicted genes was 3,346, of which 3,236 encoded proteins and 110 encoded RNAs. The majority of the protein-coding genes (78.12%) were assigned a putative function, while the remaining ones were annotated as hypothetical proteins. In total we assigned 2,337 (69.84%) of the genes. Figure 3 shows the distribution of genes into functional categories of clusters of orthologous groups (COGs). There are some indications of the presence of a prophage in the genome of strain 8N4^T^. The prophage genes are presented in Fig. 4 and listed in Table S1. Some of the traits that were found in the genome of *R. nectarea* strain 8N4^T^ are listed in Table S2.

**Table 3:** General statistics for the genome of *R. nectarea* strain 8N4^T^.

Attribute	Value	% of Total
Genome size (bp)	3,294,717	100.00
DNA coding (bp)	2,908,564	88.28
DNA GC (bp)	1,561,061	47.38
DNA scaffolds	30	100.00
Total genes	3,263	100.00
Protein-coding genes	3,073	96.71
RNA genes	110	3.29
Genes in internal clusters	605	18.08
Genes with function prediction	2,614	78.12
Genes assigned to COGs	2,337	69.84
Genes with Pfam domains	2,763	82.58
Genes with signal peptides	270	8.07
Genes with transmembrane proteins	703	21.01

**Note:**

COGs, clusters of orthologous groups; Pfam, a database of protein families.

## Discussion

### Insights from the genome sequencing of strain 8N4^T^

*Rosenbergiella nectarea* strain 8N4^T^ is a plant associated bacteria. Some genes found in this species’ genome are virulence genes that reveal the potential of this species to attack plant tissues. For example, *R. nectarea* strain 8N4^T^ possesses two genes: pectate lyase and pectin lyase fold/virulence factor. Pectin is an oligosaccharide of the plant cell wall. Pectin and pectate lyase are virulence factors that degrade the pectic components of the plant cell wall into oligosaccharides (Hugouvieux-Cotte-Pattat, Condemine & Shevchik, 2014) (Table S2). The genome of *R. nectarea* strain 8N4^T^ also encodes five hemolysin genes: hemolysin, which contains CBS domains (Radulovic et al., 1999); hemolysin III; hemolysin-activation/secretion protein; putative hemolysin; MarR family transcriptional regulator, and transcriptional regulator for hemolysin. The virulence factor hemolysin has a lytic activity on eukaryotic cells. Three copies of a gene encoding filamentous hemagglutinin family N-terminal domain are encoded in the genome of strain 8N4^T^, representing another virulence trait of this bacterium. The filamentous, hemagglutinin-like family of adhesin genes includes very long proteins from a number of plant and animal pathogens (Rojas et al., 2002). Pili, fimbriae or flagella also have a role in adhering the bacteria to its host and, thus, are also considered as virulence factors (Sauer et al., 2000). The potential of *R. nectarea* strain 8N4^T^ to produce pili and fimbriae is evident from the presence of the following genes: (i) major type-1 subunit fimbrin (pilin), (ii) prepilin-peptidase-dependent protein D, (iii) minor fimbrial subunit and (iv) fimbrial-chaperone protein. The existence of flagella is indicated by the presence of the gene c-di-GMP-binding flagellar-brake protein YcgR, which contains PilZNR and PilZ domains (Table S2).

An interesting feature found in the genome of *R. nectarea* strain 8N4^T^ is genes for S-type pyocin production (Table S2). Pyocins are polypeptide toxins that have antibacterial activity. S-type pyocins cause cell death by DNA breakdown due to endonuclease activity (Michel-Briand & Baysse, 2002). A bacterium’s production of antibiotics that act to inhibit other bacterial species is an advantage when they compete with other species in the same habitat (e.g., floral nectar).

Another noteworthy feature that can be observed in the whole genome sequence of *R. nectarea* strain 8N4^T^, is the presence of 44 genes of a prophage. There are some indications in the genes listed in Table S1 that this may be a P2-like prophage. P2 has a double-strand linear DNA molecule, an icosahedral capsid and a contractile tail and is found in members of the *Enterobacteriaceae* family (Christie & Calendar, 2016) (Fig. 4; Table S1).

Fridman et al. (2012) hypothesized that bacteria that inhabit nectar may modify their chemical compositions; for example, bacteria in the nectar may produce volatiles that may affect flower visitors. Subsequently, Rering et al. (2017) demonstrated that microbial inhabitants of floral nectar produce different volatiles that may influence visitation of a generalist pollinator. Interestingly, the genome of *R. nectarea* strain 8N4^T^ demonstrates that this species has the potential to produce different volatiles that may act in different ways on plant visitors. The presence of carbamate kinase may indicate that the bacteria produce a volatile repellant, because the function of carbamate kinase is allantoin catabolism to oxamate and carbamoyl phosphate. Allantoin was reported as a chemical compound in floral nectar (Kevan & Baker, 1983) and oxamate (the product of allantoin degradation by carbamate kinase) was reported as an insect repellant (Shatskaya, 1987; Mosin, Turlakov & Mikhal’chenkov, 2000).

By using the antiSMASH database (Blin et al., 2019), we found that *R. nectarea* strain 8N4^T^ has the potential to produce terpenes. The core biosynthetic genes for terpene biosynthesis that were found in strain 8N4^T^ are lycopene beta-cyclase and phytoene synthase. Terpenes are volatiles that can deter herbivores and attract pollinators (Perveen & Al-Taweel, 2018). The presence of the gene for squalene/phytoene synthase implies that *R. nectarea* strain 8N4^T^ has the potential to produce squalene—an insect attractant (Dutton et al., 2002; Jones et al., 2011) (Table S1).

## Conclusions

The genome of *R. nectarea* strain 8N4^T^ suggests potential roles that this species may play in the plant. On the one hand, it encodes virulence genes, like pectin lyase, hemolysin, pili and fimbria, that may cause damage to the plant tissue. On the other hand, it contains genes for producing volatile compounds, like squalene and terpenes, which may attract pollinators, as well as oxamate and terpenes, which may deter plant herbivores and nectar robbers like thrips. *R. nectarea* strain 8N4^T^ also has the potential to produce the antibiotic pyocin that enables it to thrive in its nectar habitat. Thus, we hypothesize that *R. nectarea* may have mutualistic interactions with the plant host and may affect plant pollination and fitness. However, more research is needed to confirm this hypothesis.

## Supplemental Information

10.7717/peerj.8822/supp-1 Supplemental Information 1 A list of 44 genes found in the genome sequence of *R. nectarea* strain 8N4^T^ that indicate the presence of a prophage (probably a P2-like prophage).

10.7717/peerj.8822/supp-2 Supplemental Information 2 Features of the genome of *R. nectarea* strain 8N4^T^.

## Additional Information and Declarations

### Data Availability

The following information was supplied regarding data availability: The Whole Genome Shotgun project is available at NCBI ENA: FOGC01000001–FOGC01000030 and at NCBI IMG: GCF_900111105.1.
