# Supplementary material for: Draft genome of Rosenbergiella nectarea strain 8N4T provides insights into the potential role of this species in its plant host
Source: PeerJ. 2020 Apr 6;8:e8822. doi: 10.7717/peerj.8822 (PMC7144588; doi:10.7717/peerj.8822)
Supplement: Supplemental Information 1 [file peerj-08-8822-s001.docx]

**Table S1.** **A list of 44 genes found in the genome sequence of *R. nectarea* strain 8N4^T^ that indicate the presence of a prophage (probably a P2-like prophage)**

| **Gene ID** | **Locus Tag** | **Gene Product Name** |  |
| --- | --- | --- | --- |
|  |  |  |  |
| 2654005483 | Ga0111567_10133 | 1. Phage major capsid protein, HK97 family | |
| 2654005485 | Ga0111567_10135 | 1. Phage portal protein, HK97 family | |
| 2654005486 | Ga0111567_10136 | 1. Phage head-tail adaptor, putative, SPP1 family | |
| 2654005487 | Ga0111567_10137 | 1. Uncharacterized phage protein (possible DNA packaging) | |
| 2654005490 | Ga0111567_10140 | 1. Phage terminase, small subunit, putative, P27 family | |
| 2654005491 | Ga0111567_10141 | 1. Phage terminase-like protein, large subunit, contains N-terminal HTH domain | |
| 2654005632 | Ga0111567_101183 | 1. Plasmid and phage DNA primase | |
| 2654005634 | Ga0111567_101185 | 1. Phage-integrase family protein | |
| 2654005637 | Ga0111567_101188 | 1. Toxin homologue of phage lysozyme | |
| 2654005729 | Ga0111567_101280 | 1. Replicative DNA helicase (phage and plasmid) | |
| 2654005731 | Ga0111567_101282 | 1. Phage/conjugal plasmid C-4 type zinc finger protein, TraR family | |
| 2654005906 | Ga0111567_101458 | 1. Phage-related lysozyme (muramidase), GH24 family | |
| 2654006024 | Ga0111567_1026 | 1. Phage major capsid protein, HK97 family | |
| 2654006029 | Ga0111567_10211 | 1. Bacteriophage-related protein of unknown function | |
| 2654006038 | Ga0111567_10220 | 1. Phage tail tape measure protein, lambda family | |
| 2654006039 | Ga0111567_10221 | 1. Phage-related protein | |
| 2654006043 | Ga0111567_10225 | 1. lambda-like phage minor tail protein L | |
| 2654006045 | Ga0111567_10227 | 1. Phage-related protein, tail component | |
| 2654006046 | Ga0111567_10228 | 1. Phage-related protein, tail component | |
| 2654006124 | Ga0111567_102106 | 1. Phage tail tape measure protein, TP901 family, core region | |
| 2654006125 | Ga0111567_102107 | 1. Phage tail assembly chaperone protein, E, or 41 or 14 | |
| 2654006129 | Ga0111567_102111 | 1. Phage Tail Collar Domain | |
| 2654006130 | Ga0111567_102112 | 1. Phage tail protein, **P2** protein I family | |
| 2654006131 | Ga0111567_102113 | 1. Phage-related baseplate-assembly protein | |
| 2654006133 | Ga0111567_102115 | 1. Phage baseplate assembly protein V | |
| 2654006134 | Ga0111567_102116 | 1. Phage virion morphogenesis (putative tail completion) protein | |
| 2654006135 | Ga0111567_102117 | 1. **P2** phage tail-completion protein R (GpR) | |
| 2654006136 | Ga0111567_102118 | 1. Phage lysis-regulatory protein, LysB family | |
| 2654006138 | Ga0111567_102120 | 1. Phage-related lysozyme (muramidase), GH24 family | |
| 2654006139 | Ga0111567_102121 | 1. Bacteriophage holin family HP1 | |
| 2654006140 | Ga0111567_102122 | 1. **P2**-like prophage tail protein X | |
| 2654006141 | Ga0111567_102123 | 1. Phage head-completion protein (GPL) | |
| 2654006142 | Ga0111567_102124 | 1. Phage small-terminase subunit | |
| 2654006143 | Ga0111567_102125 | 1. Phage major capsid protein, **P2** family | |
| 2654006144 | Ga0111567_102126 | 1. Phage capsid-scaffolding protein (GPO) serine peptidase | |
| 2654006146 | Ga0111567_102128 | 1. Phage portal protein, PBSX family | |
| 2654006153 | Ga0111567_102135 | 1. Bacteriophage replication gene A protein (GPA) | |
| 2654006156 | Ga0111567_102138 | 1. Phage/conjugal plasmid C-4 type zinc finger protein, TraR family | |
| 2654006160 | Ga0111567_102142 | 1. Phage regulatory protein CII (CP76) | |
| 2654006162 | Ga0111567_102144 | 1. Bacteriophage CI-repressor helix-turn-helix domain-containing protein | |
| 2654006414 | Ga0111567_10331 | 1. Phage shock protein A (PspA) family protein | |
| 2654006415 | Ga0111567_10332 | 1. Phage shock protein B | |
| 2654006416 | Ga0111567_10333 | 1. Phage shock protein C (PspC) family protein | |
| 2654006417 | Ga0111567_10334 | 1. Phage shock protein D | |
