# Supplementary material for: Draft genome of Rosenbergiella nectarea strain 8N4T provides insights into the potential role of this species in its plant host
Source: PeerJ. 2020 Apr 6;8:e8822. doi: 10.7717/peerj.8822 (PMC7144588; doi:10.7717/peerj.8822)
Supplement: Supplemental Information 2 [file peerj-08-8822-s002.docx]

**Table S2. Features of the genome of *R. nectarea* strain 8N4^T^.**

| **Gene ID** | **Locus Tag** | **Gene Product Name** |
| --- | --- | --- |
|  |  | **Plant virulence factors** |
|  |  | **Pectic-components degradation** |
| 2654006175 | Ga0111567_102157 | Pectin lyase |
| 2654006186 | Ga0111567_102168 | Pectate lyase |
|  |  | **Hemolysin** |
| 2654005796 | Ga0111567_101347 | Hemolysin, contains CBS domains |
| 2654006344 | Ga0111567_102327 | Hemolysin III |
| 2654006382 | Ga0111567_102365 | Hemolysin-activation/secretion protein |
| 2654006433 | Ga0111567_10350 | Putative Hemolysin |
|  |  | **Adhesion** |
| 2654006383 | Ga0111567_102366 | Filamentous hemagglutinin |
| 2654005459 | Ga0111567_1019 | Major type-1 subunit fimbrin (pilin) |
| 2654005951 | Ga0111567_101503 | Prepilin peptidase-dependent protein D |
| 2654005458 | Ga0111567_1018 | Minor fimbrial subunit |
| 2654006220 | Ga0111567_102202 | Fimbrial-chaperone protein |
| 2654005681 | Ga0111567_101232 | c-di-GMP-binding flagellar-brake protein YcgR, contains PilZNR and PilZ domains |
|  |  | **Volatiles production** |
| 2654005603 | Ga0111567_101153 | Carbamate kinase (insect repellent) |
| 2654006197 | Ga0111567_102179 | Phytoene desaturase (insect attractant) |
| 2654006198 | Ga0111567_102180 | Phytoene synthase (insect attractant) |
| antiSMASH database |  | Phytoene synthase together with lycopene beta-cyclase (terpene) |
|  |  | **Stress response** |
|  |  | **Antibiotic resistance** |
| 2654005619 | Ga0111567_101169 | Multiple antibiotic-resistance protein |
| 2654006061 | Ga0111567_10243 | Multidrug resistance protein MdtO |
| 2654005690 | Ga0111567_101241 | MFS transporter, DHA1 family, bicyclomycin /chloramphenicol-resistance protein |
| 2654006060 | Ga0111567_10242 | Membrane fusion protein, multidrug efflux system |
| 2654006062 | Ga0111567_1024 | Outer membrane protein, multidrug efflux system |
|  |  | **Acid stress** |
|  | Ga0111567_10192 | Acid stress-induced BolA-like protein IbaG/YrbA |
|  |  | **Osmotic stress** |
| 2654005830 | Ga0111567_101381 | Choline dehydrogenase |
| 2654005833 | Ga0111567_101384 | Choline/glycine/proline-betaine transport protein |
| 2654005831 | Ga0111567_101382 | Betaine aldehyde dehydrogenase |
| 2654005460 | Ga0111567_10110 | MFS transporter, MHS family, proline/betaine transporter |
| 2654006222 | Ga0111567_102204 | MFS transporter, MHS family, proline/betaine transporter |
| 2654005813 | Ga0111567_101364 | Osmotically inducible protein OsmY, contains BON domain |
| 2654006268 | Ga0111567_102251 | Hyperosmotically inducible protein |
| 2654006404 | Ga0111567_10321 | Osmotically inducible lipoprotein OsmB |
| 2654005908 | Ga0111567_101460 | Miniconductance mechanosensitive channel (protection against hypoosmotic shock) |
|  |  | **Oxidative stress** (antioxidants) |
| 2654005911 | Ga0111567_101463 | Peroxiredoxin (alkyl hydroperoxide reductase subunit C) |
| 2654006428 | Ga0111567_10345 | Thiol peroxidase (atypical 2-Cys peroxiredoxin) |
|  |  | [***Carotenoid biosynthesis***](https://img.jgi.doe.gov/cgi-bin/w/main.cgi?section=KeggMap&page=keggMap&map_id=map00906&gene_oid=2654006196&myimg=0) |
| 2654006196 | Ga0111567_102178 | Lycopene beta-cyclase |
| 2654006199 | Ga0111567_102181 | Beta-carotene 3-hydroxylase |
|  |  | **Siderophores** (high-affinity iron-chelating compounds) |
| 2654005559 | Ga0111567_101109 | Enterobactin synthetase component F |
| 2654005561 | Ga0111567_101111 | Ferric enterobactin receptor |
| 2654005829 | Ga0111567_101380 | Catecholate siderophore receptor |
| antiSMASH database | Ga0111567_109 | Siderophore synthetase component ([Desferrioxamine](https://www.google.com/search?q=desferrioxamine&spell=1&sa=X&ved=2ahUKEwi7hp3quZLnAhUD2KQKHRqWDJcQkeECKAB6BAgOECY)) |
|  |  | **Antibiotics** |
| 2654006384 | Ga0111567_1031 | S-type Pyocin (protein antibiotics) |
| 2654006387 | Ga0111567_1034 | S-type Pyocin |
